# Surgical treatment and outcomes in feline aortic thromboembolism: a retrospective study of 13 cats

**DOI:** 10.3389/fvets.2025.1618435

**Published:** 2025-10-15

**Authors:** Jin Li, Yuhu Zhou, Yanhai Liu, Qingbo Ma, Jingwen Peng, Meng Li

**Affiliations:** ^1^Department of Veterinary Clinical Sciences, College of Veterinary Medicine, Nanjing Agricultural University, Nanjing, Jiangsu, China; ^2^AMC Riverside Referral Center Animal Hospital, Nanjing, Jiangsu, China; ^3^Ai-bi Animal Medical Center, Nanjing, Jiangsu, China; ^4^Nanjing Agricultural University Veterinary Teaching Hospital, Nanjing, Jiangsu, China

**Keywords:** surgical aortic thrombectomy, thrombosis, survival-to-discharge rate, azotemia, neutrophil-to-lymphocyte ratio

## Abstract

**Objectives:**

This study aimed to evaluate the survival and survivor characteristics in cats diagnosed with feline aortic thromboembolism (FATE) who underwent surgical aortic thrombectomy.

**Methods:**

Medical records from 2021 to 2023 were retrospectively reviewed for cats diagnosed with FATE that underwent surgical aortic thrombectomy. Data collected included signalment, medical history, clinical examination findings, laboratory parameters before and after surgery, the time from FATE onset to surgery, treatments administered, survival to discharge, and recurrence or long-term outcomes in discharged cats.

**Results:**

Thirteen client-owned cats met the inclusion criteria. Common postoperative laboratory abnormalities observed during hospitalization included azotemia (*n* = 8), anemia (*n* = 4), hyperkalemia (*n* = 4), and elevated alanine aminotransferase (*n* = 3). After surgery, 53.8% of the cats survived to discharge, with 71.4% showing complete recovery of hind limb motor function. Among the discharged cats, two (28.6%) were confirmed deceased during follow-up, while five (71.4%) were lost to follow-up. The median follow-up duration was 37 days (14–498). Recurrence of FATE occurred in two cats (28.6%) at 77 and 493 days postoperatively; both were successfully managed with medical treatment and survived to discharge again. Cats that survived had significantly lower preoperative neutrophil-to-lymphocyte ratios (*p* = 0.033), postoperative serum potassium levels (*p* = 0.037), and postoperative blood urea nitrogen concentrations (*p* = 0.037) than non-survivors.

**Conclusion and relevance:**

Cats undergoing surgical aortic thrombectomy for FATE showed a 53.8% survival rate to discharge, with 71.4% of survivors achieving full recovery of limb motor function. Surgical aortic thrombectomy may be considered as a treatment option for cats with FATE, particularly when timely presentation allows for early intervention.

## 1 Introduction

Feline aortic thromboembolism (FATE) occurs when an intracardiac thrombus dislodges and enters the systemic arterial circulation, resulting in arterial obstruction and acute ischemia of downstream tissues (1, 2). It is often secondary to cardiomyopathies such as hypertrophic cardiomyopathy (HCM), hypertrophic obstructive cardiomyopathy (HOCM), dilated cardiomyopathy (DCM), and restrictive cardiomyopathy (3, 4). Other contributing factors include hyperthyroidism and neoplasia (3, 5). Thrombi typically originate in the left atrium and may dislodge into the systemic circulation (6), most frequently lodging at the aortic trifurcation or, less commonly, in vessels such as the renal, cerebral, mesenteric, or brachial arteries (7). FATE symptoms develop acutely and often progress rapidly, necessitating prompt diagnosis and intervention.

Initial diagnosis is based on the “5 Ps”: pain, pulselessness, paralysis, polar, and pallor (4). Management strategies include antithrombotic therapy, thrombolytic therapy, pain control, treatment of underlying cardiac disease, and supportive care (4, 7, 8). Anticoagulants (e.g., heparin and rivaroxaban) and antiplatelet agents (e.g., aspirin and clopidogrel) are commonly used, often in combination as dual therapy (4, 9, 10). Research on thrombolytic therapy in FATE remains limited. The most comprehensive prospective controlled study to date reported that tissue plasminogen activator (TPA) improved limb function but did not confer a survival benefit (2). Since cats with FATE often present with congestive heart failure (CHF) and advanced cardiomyopathy, the risk of anesthesia-related complications is considerable, and conservative medical management remains the predominant approach in clinical practice. Surgical intervention has been described only in isolated case reports (11–17), and evidence regarding clinical parameters or survival characteristics in cats with FATE remains limited. These gaps underscore the need for additional research to better guide clinical decision-making.

The present study retrospectively evaluated 13 cats diagnosed with FATE that underwent surgical aortic thrombectomy. The objectives were to assess postoperative outcomes, such as clinical recovery, survival-to-discharge, and recurrence during the follow-up period, and to evaluate the characteristics of cats that survived to discharge, thereby addressing the current knowledge gap regarding surgical aortic thrombectomy in FATE management.

## 2 Materials and methods

### 2.1 Case selection and medical record review

A retrospective study was conducted to review cases of FATE at the Ai-bi Animal Medical Center Hospital and the AMC Riverside Referral Center Animal Hospital in Nanjing, China, between February 2021 and February 2023. The inclusion criteria for study entry were cats with a clinical or definitive diagnosis of FATE that underwent surgical aortic thrombectomy. Fourteen cats initially met the inclusion criteria; however, one cat with paralysis of all four limbs due to FATE was excluded, as the prognosis was considered grave and surgical intervention was unlikely to provide clinical benefit, particularly for the forelimb deficits. In this study, the clinical diagnosis of FATE was primarily based on clinical signs, such as pale or cyanotic paw pads, decreased limb temperature, pain, and absence of a palpable arterial pulse in affected limbs (7). Definitive diagnosis of FATE was based on imaging findings, with abdominal ultrasound used to assess the loss of blood flow in the abdominal aorta and/or renal arteries and contrast-enhanced computed tomography (CT) performed to localize the site of thromboembolism.

The collected data included age, sex, breed, weight, history, clinical symptoms, physical examination findings, laboratory results, diagnostic imaging (echocardiography, abdominal ultrasonography, thoracic radiography, and CT), interval from FATE onset to surgery, anesthesia protocols, treatments, and clinical outcomes.

Laboratory parameters (complete blood count, serum biochemistry, and blood gas analysis) at presentation and on postoperative day 1 were recorded and compared between cats that survived to discharge and those that died during hospitalization. Anemia was defined as a hematocrit (HCT) of <30.3% at diagnosis. Hyperkalemia was defined as blood potassium concentrations of ≥4.20 mmol/L, and azotemia was defined as blood urea nitrogen (BUN) of ≥34.0 mg/dL or creatinine of ≥2.40 mg/dL (18). These cutoffs were based on the reference intervals of IDEXX (for HCT and creatinine) and Abbott (for BUN, blood potassium), which provided laboratory testing for the study patients.^1^^,^^2^ Restoration of arterial blood flow following thrombus removal is the goal of surgery; however, reperfusion injury may occur as a result of sudden reperfusion of the ischemic tissue, which can lead to the release of potassium and organic acids, potentially leading to life-threatening hyperkalemia and metabolic acidosis (10, 19, 20).

CHF in cats was diagnosed based on one or more of the following criteria: clinical signs (tachypnea, labored breathing, and pulmonary crackles); thoracic radiographs showing pulmonary infiltrates and cardiomegaly, consistent with cardiogenic pulmonary edema; and ultrasonography demonstrating pleural effusion or B-lines with left atrial enlargement (21).

For cats discharged from the hospital, information was collected on post-discharge medications, recurrence of FATE, and treatments administered following recurrence. For cats that died after discharge, survival time and cause of death were recorded. Survival time was defined as the interval from initial presentation to death, including both humane euthanasia and spontaneous cardiopulmonary arrest. Follow-up time was defined as the interval from the initial presentation to the last recorded follow-up. Cases were censored if the cats were lost to follow-up, were alive at the end of follow-up, had an unknown death date, or died from unrelated causes. For cases lost to follow-up, censoring was based on the date of the last client–veterinarian contact. The final follow-up date was 27 March 2025.

### 2.2 Statistical analysis

Statistical analysis was carried out using SPSS (version 30.0, IBM Corp., CA, United States). Cats were categorized by short-term outcomes as either surviving to discharge or dying during hospitalization; the latter included both spontaneous cardiopulmonary arrest and humane euthanasia. The normality of continuous variables was assessed using the Shapiro–Wilk test. Due to the small sample size, all continuous variables were expressed as median (minimum-maximum), irrespective of their distribution. For continuous variables, independent samples *t*-tests were used to assess short-term outcomes when data were normally distributed, and the Mann–Whitney U tests were used when data were non-normally distributed. The independent variables analyzed included age, body weight, rectal temperature, neutrophil-to-lymphocyte ratio (NLR), blood potassium, BUN, creatinine, and HCT. NLR, a marker of systemic inflammation and stress, has been linked to prognosis in both human and feline hypertrophic cardiomyopathy. Laboratory parameters on postoperative day 1 were analyzed to assess differences between cats that survived to discharge and those that died during hospitalization. The associations of the interval from FATE onset to surgery (≤4 h vs. >4 h), postoperative hyperkalemia, postoperative elevated BUN, and postoperative NLR with short-term outcomes were analyzed using Fisher’s exact test. A *p*-value of <0.05 was considered statistically significant.

## 3 Results

### 3.1 Animals and clinical signs

A total of 13 cats met the inclusion criteria for this study. The cats had a median age of 3.42 years (1.58–7.17), a body weight of 5.00 kg (2.90–6.30), and a rectal temperature of 38.20 °C (37.20–39.00). Among the 13 cats, 9 were male (69.2%) and 4 were female (30.8%). The breeds included British shorthair (*n* = 6), Domestic shorthair (*n* = 2), mixed breed (*n* = 2), American shorthair (*n* = 1), Ragdoll (*n* = 1), and Munchkin cat (*n* = 1). In this study population, cats that did not survive to discharge had similar ages (*p* = 0.882), body weights (*p* = 0.408), and rectal temperatures (*p* = 0.292) compared to those that survived.

The most common clinical signs observed were limb hypothermia (11/13, 84.6%), hind limb pain (9/13, 69.2%), and hind limb paralysis (13/13, 100.0%). One cat (7.7%) showed lameness and decreased skin temperature in the right forelimb before the onset of FATE but recovered spontaneously without treatment. The remaining 12 cats had no relevant medical history. None of the cats had received any medications within the past year.

### 3.2 Diagnostic evaluation

Seven cats were diagnosed with FATE based solely on clinical symptoms, without further imaging (cases 1, 2, 5, 6, 9, 11, and 13). An additional three cats were diagnosed via abdominal ultrasound (cases 3, 4, and 8), among which two cats (cases 3 and 4) had thrombi detected in the abdominal aorta, although the thrombus location for case 8 was not specified in the report. Three cats were diagnosed using contrast-enhanced CT: two (cases 7 and 12) with thrombi at the aortic bifurcation and one (case 10) with a thrombus in the distal abdominal aorta, immediately proximal to the aortic bifurcation. Among the cats that underwent abdominal ultrasound or contrast-enhanced CT, only case 8 showed right renal atrophy with associated regional perfusion abnormalities; no renal artery thrombi or renal perfusion abnormalities were reported in the other cases.

Preoperative echocardiographic records were available for 11 cats, of which one cat (case 9) showed no abnormalities (Table 1). However, this cat was not evaluated for hyperthyroidism or neoplasia; thus, underlying conditions potentially associated with FATE could not be ruled out. Among the remaining cats, eight (72.7%) were diagnosed with HCM, one (9.1%) with HOCM, nine (81.8%) had severe left atrial enlargement, and four (36.4%) exhibited spontaneous echocardiographic contrast (SEC) on echocardiography.

**Table 1:** Clinical and surgical characteristics of 13 cats with FATE.

Cat	Underlying disease	CHF	The duration of the operation	Onset of FATE to surgical time	Anesthetic protocol
1	HOCM, SEC, LA enlargement	Yes	45.0 min	>12.0 h	Butorphanol, propofol, and isoflurane
2	LA enlargement	Yes	47.0 min	11.0 h	Propofol and isoflurane
3	HCM, SEC, LA enlargement	—	—	3.5 h	Butorphanol, propofol, and isoflurane
4	HCM, LA enlargement, SEC, cardiomyopathy	Yes	—	>12.0 h	Butorphanol, propofol, and isoflurane
5	HCM, LA enlargement	—	67.0 min	—	Propofol and isoflurane
6	—	—	55.0 min	—	Propofol and isoflurane
7	—	—	40.0 min	3.0 h	Butorphanol, propofol, and isoflurane
8	HCM, LA enlargement	—	55.0 min	>12.0 h	Butorphanol, propofol, and isoflurane
9	—	No	40.0 min	7.5 h	Butorphanol, propofol, and isoflurane
10	HCM, LA enlargement	Yes	55.0 min	—	Butorphanol, propofol, and isoflurane
11	HCM, LA enlargement	Yes	34.0 min	—	Butorphanol, propofol, and isoflurane
12	HCM, LA enlargement	Yes	42.0 min	2.5 h	Butorphanol, propofol, and isoflurane
13	HCM, SEC	No	85.0 min	>6.0 h	Butorphanol, propofol, and isoflurane

FATE, feline aortic thromboembolism; CHF, congestive heart failure; HOCM, hypertrophic obstructive cardiomyopathy; SEC, spontaneous echocardiographic contrast; LA, left atrial; HCM, hypertrophic cardiomyopathy.

—, No relevant information was recorded.

At initial presentation, CHF was present in six cats, absent in two, and unknown in five (Table 1).

### 3.3 Treatment

All 13 cats underwent surgical aortic thrombectomy. Prior to the induction of general anesthesia, cats presenting with pulmonary edema received intravenous furosemide (2 mg/kg). Butorphanol (0.2 mg/kg, IV) was administered for analgesia. Anesthesia was induced with intravenous propofol (4 mg/kg, to effect) and maintained with isoflurane. The surgical procedure was conducted in accordance with techniques described in previous studies (16, 17). Briefly, under general anesthesia, a standard caudal celiotomy was performed to expose the abdominal aorta, allowing identification of the thrombus. The obstructed aorta was bluntly dissected from the surrounding tissues, and arterial clamps (Tigergene Co., Ltd., China) or tourniquets (EM-SB1, Shenzhen Cement Co., Ltd., China) were placed cranially and caudally to the thrombus. An arteriotomy was performed to extract the thrombus. Luminal flushing with diluted enoxaparin sodium (70 IU/kg, Hangzhou Jiuyuan Genetic Biopharmaceutical Co., Ltd., China) was conducted in cases 2, 5, 7, 8, 9, 10, and 12, whereas luminal flushing was not performed in the remaining cases. The arterial incision was closed using a continuous suture pattern. The suture material varied among surgeons, with either 8–0 absorbable braided PGA sutures or 6–0 absorbable monofilament PDO sutures employed. Arterial clamps or tourniquets were released sequentially, starting with the caudal clamp or tourniquet, to restore blood flow. Once hemostasis was confirmed and arterial pulsation had resumed, the abdominal cavity was closed using standard procedures. Postoperatively, all cats were maintained in an oxygen chamber with an oxygen concentration of 40%.

Medications administered during the postoperative hospitalization and after discharge are summarized in Table 2. Postoperative medical management primarily included antithrombotic therapy, cardiac medications, pain management, and supportive treatment for ischemia–reperfusion injury. Among the seven cats that survived to discharge, all received antithrombotic therapy, and six received cardiac medications for the underlying heart disease. Two cats received lappaconitine hydrobromide (0.4 mg/kg, IM, q24h) postoperatively for analgesia.

**Table 2:** Treatments, clinical outcomes, and follow-up in 13 cats with FATE.

Cat	Medications during hospitalization	Medications after discharge	Motor function recovery status	Recurrence	Clinical outcome	Survival or follow-up time (days)
1	Low molecular weight heparin; clopidogrel; pimobendan; furosemide; lappaconitine hydrobromide; butorphanol; ulinastatin; diphenhydramine; compound amino acid; 0.9% sodium chloride	Rivaroxaban; clopidogrel; pimobendan; furosemide; and diltiazem	Partial recovery	Recurrence of ATE 493 days after surgery	Survival to discharge but lost to follow-up	498
2	Clopidogrel; benazepril; furosemide; glutathione; vitamin B complex; 0.9% sodium chloride	Clopidogrel and benazepril	Recovered	No	Survival to discharge, but was euthanized during follow-up	14
3	Low molecular weight heparin; clopidogrel; dopamine; furosemide; butorphanol; vitamin B complex; vitamin C; compound amino acid; lactated Ringer’s solution	Clopidogrel; pimobendan; and furosemide	No	No	Survival to discharge, but lost to follow-up	30
4	Pimobendan; butorphanol; furosemide; vitamin B complex; vitamin C	None	—	Died	Spontaneous cardiopulmonary arrest during postoperative hospitalization	3
5	Vitamin B complex; lactated Ringer’s solution	None	—	Died	Spontaneous cardiopulmonary arrest during postoperative hospitalization	3
6	Clopidogrel; pimobendan; dobutamine; furosemide; lappaconitine hydrobromide; butorphanol; vitamin B complex; compound amino acid; lactated Ringer’s solution	None	No	Died	Spontaneous cardiopulmonary arrest during postoperative hospitalization	12
7	Clopidogrel; butorphanol; vitamin B complex; vitamin C; compound amino acid; lactated Ringer’s solution	Low molecular weight heparin and clopidogrel	Recovered	No	Survival to discharge but lost to follow-up	33
8	Clopidogrel; benazepril; furosemide; butorphanol; lactated Ringer’s solution	Clopidogrel; benazepril; furosemide; and pimobendan	Recovered	No	Survival to discharge but died during follow-up	439
9	Low molecular weight heparin; clopidogrel; butorphanol; glutathione; vitamin B complex; vitamin C; lactated Ringer’s solution	None	No	Died	Spontaneous cardiopulmonary arrest during postoperative hospitalization	1
10	Clopidogrel; benazepril; furosemide; butorphanol; glutathione; vitamin B complex; vitamin C; lactated Ringer’s solution	None	No	Died	Euthanized after surgery	7
11	Low molecular weight heparin; clopidogrel; benazepril; furosemide; butorphanol; vitamin B complex; vitamin C; 0.9% sodium chloride	Clopidogrel; benazepril; and spironolactone	Recovered	Recurrence of CHF 45 days after discharge and recurrence of ATE 77 days after surgery	Survival to discharge but lost to follow-up	103
12	Clopidogrel; benazepril; furosemide; butorphanol; meloxicam; vitamin B complex; vitamin C; lactated Ringer’s solution	Clopidogrel; benazepril; and furosemide	Recovered	No	Survival to discharge but lost to follow-up	37
13	Low molecular weight heparin; clopidogrel; benazepril; butorphanol; glutathione; vitamin B complex; vitamin C; lactated Ringer’s solution	None	No	Died	Spontaneous cardiopulmonary arrest during postoperative hospitalization	1

—, No relevant information was recorded.

### 3.4 Outcome

#### 3.4.1 Clinical outcomes

Detailed information for each cat, such as the time from the onset of FATE to surgery, recurrence, and outcomes, is provided in Tables 1, 2. Common postoperative laboratory abnormalities observed during hospitalization included azotemia (*n* = 8), anemia (*n* = 4), hyperkalemia (*n* = 4), and elevated alanine aminotransferase (*n* = 3). Among the 13 cats, 6 (46.2%) died during postoperative hospitalization, including 1 cat (case 10) that was euthanized due to poor prognosis based on clinical evaluation and the owner’s decision, and 5 cats died of spontaneous cardiopulmonary arrest, presumed to be secondary to complications of FATE. None of these cats regained bilateral hind limb pulses or motor function after surgery.

The recovery status of limb motor function was available in 11 cats (Table 2). Of the seven cats that survived to discharge, five cats (cases 2, 7, 8, 11, 13) achieved complete recovery of hind limb motor function. Case 1 regained motor function in the left hind limb but continued to exhibit right hind limb deficits, characterized by dragging and absent voluntary movement. At a 3-month postoperative follow-up, partial recovery of proprioception in the right hindlimb was observed, although reflexes remained weak. Case 3 showed no improvement in hindlimb motor function 30 days postoperatively and subsequently developed bilateral hindlimb edema.

The median follow-up duration for cats that survived to discharge was 37 days (14–498 days; Table 2). Seven cats were censored at their last recorded follow-up. Two cats (cases 2 and 8) were confirmed deceased, while the remaining five were lost to follow-up. Case 2 was euthanized 14 days postoperatively due to feline parvovirus infection. Case 8 developed left atrial thrombosis and mild pulmonary edema 7 months postoperatively, and its last follow-up was recorded 439 days after surgery.

FATE recurrence occurred in two cats. Case 1 was readmitted with recurrence of FATE 493 days after surgery, recovered after medical treatment, and was discharged 5 days later, but was subsequently lost to follow-up. The treatment included enoxaparin sodium (100 IU/kg, SC, q12h), clopidogrel (18.75 mg, PO, q24h), furosemide (1 mg/kg, SC, q12h), pimobendan (0.125 mg/kg, PO q12h), dobutamine (2–5 μg/kg/min, CRI), and lappaconitine hydrobromide (0.4 mg/kg, IM, q24h). Case 11 was hospitalized for the recurrence of FATE at 77 days postoperatively and was managed with conservative treatment. Treatment included enoxaparin sodium (100 IU/kg, SC, q24h), clopidogrel (18.75 mg, PO, q24h), furosemide (1–2 mg/kg, SC, q12h), ornidazole (15 mg/kg, SC, q24h), and cefquinome sulfate (5 mg/kg, SC, q24h). Blood flow and function in both hind limbs were restored; the cat was discharged again but lost to follow-up 21 days later. No recurrence of FATE was observed in the other five cats.

#### 3.4.2 Characteristics associated with non-survival

Cats that did not survive to discharge had significantly higher preoperative NLR (12.68, 3.84–27.49) compared to those that survived (1.36, 0.69–3.93; *p* = 0.033; Table 3). When stratified by the median NLR, the survival-to-discharge rate was 20% in the high-NLR group and 80% in the low-NLR group (*p* = 0.206, Table 4).

**Table 3:** Perioperative clinical and laboratory variables in cats with FATE: comparison between survivors and non-survivors.

Classification	*n*	Reference interval	Survived to discharge	Died during hospitalization	*p*-value
The duration of the operation (min)	11	—	43.5 (34.0–55.0)	55.0 (40.0–85.0)	0.054
Preoperative neutrophil-to-lymphocyte ratio	11	—	1.36 (0.69–3.93)	12.68 (3.84–27.49)	0.033
Preoperative blood potassium (mmol/L)	11	2.90–4.20	3.80 (3.00–4.00)	3.75 (3.30–4.80)	0.455
Preoperative blood urea nitrogen (mg/dL)	11	15.00–34.00	26.00 (16.00–47.00)	25.00 (18.00–91.00)	0.293
Preoperative creatinine (mg/dL)	9	0.80–2.40	1.20 (0.95–1.84)	1.34 (0.54–5.18)	0.496
Preoperative hematocrit (%)	10	30.30–52.30	38.20 (37.10–47.30)	36.70 (33.70–42.50)	0.462
Postoperative blood potassium (mmol/L)^*^	11	2.90–4.20	3.40 (3.00–4.80)	4.20 (4.00–5.20)	0.037
Postoperative blood urea nitrogen (mg/dL)^*^	11	15.00–34.00	29.00 (16.00–89.00)	79.00 (35.00–109.00)	0.037
Postoperative creatinine (mg/dL)^*^	6	0.80–2.40	1.70 (1.07–6.72)	4.23 (1.28–7.17)	0.643

* Laboratory parameters on postoperative day 1 were included in the statistical analysis.

**Table 4:** Analysis of the association between preoperative NLR, postoperative hyperkalemia, elevated BUN, the interval between onset of FATE and surgery within 4 h, and survival to discharge using Fisher’s exact test.

Classification		No. of cats (survived to discharge/did not survive)	*p-*value
Preoperative NLR	High NLR (>3.88)	5 (1/4)	0.206
	Low NLR (≤3.88)	5 (4/1)	
Postoperative blood potassium concentration^*^	Hyperkalemia (≥4.20 mmol/L)	4 (1/3)	0.088
	Normokalemia (2.90–4.20 mmol/L)	7 (6/1)	
Postoperative BUN concentration^*^	Elevated BUN (≥34.00 mg/dL)	5 (1/4)	0.015
	Normal BUN (15.00–34.00 mg/dL)	6 (6/0)	
Interval from FATE onset to surgery	≤4 h	3 (3/0)	0.464
	>4 h	6 (3/3)	

NLR, neutrophil-to-lymphocyte ratio; BUN, blood urea nitrogen; FATE, feline aortic thromboembolism.

* Laboratory parameters on postoperative day 1 were included in the statistical analysis.

Serum biochemistry results on postoperative day 1 were available for 11 cats. Cats that survived had significantly lower postoperative BUN concentrations (29.00 mg/dL, 16.00–89.00 mg/dL) than non-survivors (79.00 mg/dL, 35.00–109.00 mg/dL; *p* = 0.037, Table 3). Cats that survived also had lower postoperative blood potassium concentrations (3.40 mmol/L, 3.00–4.80 mmol/L) than non-survivors (4.20 mmol/L, 4.00–5.20 mmol/L; *p* = 0.037, Table 3). Postoperative creatinine concentrations (*n* = 6) did not differ significantly between survivors and non-survivors (*p* = 0.643, Table 3). The survival-to-discharge rate was lower (20.0%) among cats with postoperative BUN concentrations >34.00 mg/dL (*n* = 5) than those within the reference interval (15.00–34.00 mg/dL; *n* = 6), all of which survived to discharge (*p* = 0.015; Table 4). The survival-to-discharge rate was lower in cats with postoperative hyperkalemia than in those without (*p* = 0.088, Table 4).

Among cats with available data, survival rates did not differ significantly between those that underwent surgery within 4 h of symptom onset and those that underwent surgery after 4 h (*p* = 0.464, Table 4). The median surgical duration (*n* = 11) was 47.0 min (34.0–85.0 min). Cats that survived had a shorter median surgical duration (43.5 min, 34.0–55.0 min) than cats that did not survive (55.0 min, 40.0–85.0 min; *p* = 0.054, Table 3). No anesthetic-related mortalities were observed.

## 4 Discussion

This study described 13 surgically treated FATE cats, with a survival-to-discharge rate of 53.8%. Among the cats that survived to discharge, 71.4% fully regained hindlimb motor function in both hind limbs. The median follow-up time for the discharged cats was 37 days (14–498).

Consistent with previous studies, the majority of FATE cases in this study (10/13, 76.9%) were associated with underlying cardiac disease, such as HCM, HOCM, cardiomegaly, or SEC—an echocardiographic sign of blood stasis and thromboembolic risk (22–24). Although not represented in this series, the literature indicates that a minority of FATE cases may arise secondary to neoplasia or hyperthyroidism (3, 5). Three out of 13 cats (cases 6, 7, and 9) lacked full screening, making it impossible to determine the cause. Case 9 showed no cardiac abnormalities but was not screened for hyperthyroidism or neoplasia. Case 7 was only ruled out for hyperthyroidism but was not evaluated for heart disease or neoplasia. Case 6 was not screened for heart disease, hyperthyroidism, or neoplasia. Although rare, FATE can occur without evident predisposing conditions (3, 10, 25). In such cases, when no underlying cardiac disease is identified, further targeted imaging of the affected vessels is warranted (10), and investigation for potential occult neoplasia or hyperthyroidism is also recommended.

Previous retrospective studies have reported survival-to-discharge rates for FATE ranging from 20 to 50% (3, 6, 26–28), with outcomes potentially affected by euthanasia bias. Prospective studies have documented survival-to-discharge rates of 27–45% (2, 20). In one prospective multicenter study, some veterinary practices reported discharge rates as high as 67–100%, although the number of cases was limited (2). In the present retrospective study, the overall survival-to-discharge rate was 53.8% among all surgically treated cats. Survival reached 100.0% in cats operated on within 4 h of symptom onset, compared with 50.0% in those operated on after 4 h, although this difference did not reach statistical significance (*p* = 0.464, Table 4). In people, timely intervention in acute ischemic stroke is important, with the American Heart Association/American Stroke Association guidelines recommending intravenous thrombolysis within 3 h of symptom onset, and up to 4.5 h in selected patients (29). Nevertheless, the small sample size and retrospective design of the present study preclude definitive conclusions, highlighting the need for validation through larger, multicenter prospective studies utilizing appropriate statistical methods, such as multivariate logistic regression analysis.

Among the seven cats that survived to discharge, five (71.4%) achieved complete recovery of hind limb motor function, while one (14.3%) regained motor function in the left hind limb but showed residual right hind limb paresis. Recovery timelines were inconsistently documented; however, one case recorded postoperative return of sensation and reflexes within 2 h, with full ambulation achieved within 2–3 days. Overall, among the 11 cats with available records of hind limb motor function, six (58.3%) demonstrated partial or complete recovery. When compared with previously reported outcomes with other treatments, such as TPA (45.0% survival to discharge, 25.0% limb improvement at 48 h postoperatively), placebo (30.0% survival to discharge, 20.0% limb improvement at 48 h postoperatively), enoxaparin (47.2% survival to discharge), and streptokinase (33.0% survival to discharge, 30.0% limb motor function recovery), the survival and functional recovery observed in this study appear broadly similar (2, 19, 27).

Surgical treatment for FATE remains controversial due to technical challenges, an uncertain prognosis, and the risk of severe ischemia–reperfusion injury (10). Anesthetic management is particularly challenging, as many cats present with CHF, hypotension, or shock, which complicates peri-anesthetic stabilization. In this study, six cats presented with CHF before surgery, yet none died during anesthesia. Nevertheless, both preoperative and postoperative stabilization remain difficult, as anesthesia can exacerbate cardiac dysfunction, reduce cardiac output, and precipitate hypotension and hypoxemia. Therefore, careful hemodynamic monitoring and tailored anesthetic protocols are essential. Following thrombus removal, potassium and organic acids from necrotic tissue enter the bloodstream, and reoxygenation of hypoxic tissues exacerbates cellular damage, leading to inflammation, hyperkalemia, and multi-organ failure (10, 20, 30). Such complications are an important consideration when evaluating surgical intervention; however, they may also occur with other treatment modalities. Although different veterinarians adopted individualized postoperative medication protocols, all cats received antiplatelet or Xa anticoagulant agents, diuretics, positive inotropes, analgesics, and fluid therapy, resulting in a 53.8% survival-to-discharge rate. Notably, while antiplatelet or anticoagulant therapy is necessary to reduce thrombosis risk, it may also increase the likelihood of intraoperative or postoperative hemorrhage, necessitating cautious use with close monitoring.

Regarding postoperative laboratory abnormalities, anemia was detected in four cats but was not further analyzed. In some non-anemic cats, dehydration may have masked hematocrit changes, limiting interpretation. Postoperative creatinine was measured in only six cats, and no significant difference was detected between survivors and non-survivors. The limited sample size, particularly in the non-survivor group, reduces the statistical power of this comparison. Therefore, these findings should be regarded as descriptive characteristics of non-survivors rather than validated prognostic indicators, as logistic regression could not be performed in this small cohort. Non-survivors were more likely to have elevated postoperative BUN, with four out of five cats with elevated BUN not surviving, whereas all six cats with normal BUN survived to discharge (*p* = 0.015, Table 4). This observation may reflect acute kidney injury or renal hypoperfusion, complications frequently associated with FATE and previously linked to poor outcomes (26, 27). BUN is a non-specific parameter influenced by hydration status, dietary protein intake, gastrointestinal bleeding, and renal function (31). Prospective studies with larger cohorts and standardized perioperative monitoring of creatinine and urine output are needed to clarify the prognostic significance of renal parameters.

Postoperative hyperkalemia was also observed in a subset of cats, and non-survivors had significantly higher blood potassium concentrations. Although the difference in survival-to-discharge rates between cats with and without hyperkalemia did not reach statistical significance, the trend suggests that electrolyte disturbances secondary to renal dysfunction or reperfusion injury may contribute to increased mortality. Moreover, the lack of postoperative laboratory data in two cats that died within the first days of surgery introduces survival bias and may limit the accuracy of these observations.

Cats that did not survive had a significantly higher preoperative NLR than cats that survived to discharge (*p* = 0.033). Although the survival-to-discharge was 20.0% in cats with high preoperative NLR versus 80.0% in those with low NLR, this difference did not reach statistical significance (*p* = 0.206). NLR, a known biomarker of systemic inflammation and stress, has been associated with prognosis in both human and feline HCM (30, 32–34). In cats with severe HCM, elevated NLR has been identified as an important prognostic indicator and an independent predictor of cardiac-related death (30). In a retrospective study comparing FATE cats with healthy controls, increased NLR was associated with a shorter median survival time and was suggested as a potentially valuable diagnostic biomarker for FATE (35). Similarly, numerous studies in human medicine have demonstrated the role of inflammatory mechanisms and progression of ischemic stroke (36–39). It has been suggested that NLR at admission may predict the prognosis of patients with large-vessel occlusive stroke (40), with lower NLR values associated with early neurological improvement following thrombolytic therapy (41).

Previous studies have reported that FATE recurrence is common, with rates ranging from 16.7 to 75.0% (3, 6, 8, 28, 42). In this study, among the seven cats that survived to discharge, two (28.6%) experienced recurrence at 77 days and 493 days after surgery. Both cats recovered after conservative treatment and were discharged. All surviving cats received long-term management with anticoagulants and cardiac medications. While the recurrence rate observed here is within the range reported in the literature, the small sample size and retrospective design preclude conclusions regarding the effect of surgical thrombectomy on long-term recurrence risk. Moreover, as this was a retrospective study without standardized inclusion criteria for baseline cardiac status or the presence of comorbidities such as hyperthyroidism and neoplasia, these factors may also have influenced survival and recurrence outcomes.

In this study, no significant difference in rectal temperature was observed between cats that survived to discharge and those that died during hospitalization. This contrasts with previous reports in which rectal temperature has repeatedly been identified as a prognostic indicator in cats with FATE (3, 28). This discrepancy may reflect a recruitment bias inherent in our surgical candidates, as only cats deemed clinically stable enough to undergo surgery were included, which may have limited the variability of rectal temperature in our study.

This study has several limitations. As a retrospective analysis, it was limited by incomplete clinical records and inconsistent preoperative and postoperative imaging and laboratory evaluations. In particular, the timing of the first medical treatment for the operated cats could not be consistently ascertained, which may have introduced bias in interpreting treatment outcomes. Variations in perioperative management, particularly the absence of standardized postoperative care, may also have influenced the outcomes. Importantly, cats that underwent humane euthanasia were included, and since not all deaths resulted from spontaneous cardiopulmonary arrest, the survival rate and survival time may have been underestimated. Only cats undergoing surgery were analyzed, inherently selecting for patients with greater clinical stability. Excluding cats deemed unstable for surgery introduced survivorship bias and likely overestimated prognostic outcomes relative to the broader FATE population. Due to the severity of the disease, the high proportion of humane euthanasia at presentation, and the limited number of owners opting for invasive treatment, larger cohort studies are difficult to conduct. The small sample size and loss to follow-up limited the accurate assessment of recurrence and long-term outcomes. Finally, the small sample size precluded multivariable logistic regression, restricting analyses to univariable comparisons. The limited number of events increases the risk of both Type I and Type II statistical errors, and the findings should therefore be interpreted with caution. Larger prospective studies with larger cohorts are needed to more accurately evaluate the efficacy and prognostic factors of surgical treatment for FATE.

## 5 Conclusion

We demonstrated that the survival-to-discharge rate of cats with FATE undergoing surgical aortic thrombectomy was 53.8%, which is comparable to previously reported outcomes with other treatment methods. Notably, 71.4% of surviving cats achieved full recovery of limb motor function. Significant differences were observed in preoperative NLR, postoperative blood potassium, and postoperative BUN between survivors and non-survivors. These findings may inform clinical decision-making regarding treatment strategies and euthanasia considerations, aiming to minimize suffering and improve quality of life. However, due to the small sample size and the retrospective nature of the study, the results should be interpreted with caution. Future investigations should explore surgical treatment for FATE through large-scale, multicenter prospective studies.

## Data Availability

The original contributions presented in the study are included in the article/supplementary material, and further inquiries can be directed to the corresponding authors.

## Footnotes

^1^ProCyte Dx hematology analyzer and Catalyst One chemistry analyzer (IDEXX Laboratories, Westbrook, ME, United States).

^2^i-STAT 300 handheld analyzer (Abbott Point of Care Inc., Princeton, NJ, United States).
